# Evaluation of the frequency and intensity of COVID-19 in patients with ankylosing spondylitis under anti-TNF therapy

**DOI:** 10.3906/sag-2105-69

**Published:** 2021-11-09

**Authors:** Sümeyye Merve TÜRK, Zeynep ÖZTÜRK, Damla KARATAŞ, Ünal ERKORKMAZ, Emel GÖNÜLLÜ

**Affiliations:** 1 Department of Internal Medicine, Division of Rheumatology, Faculty of Medicine, Sakarya University, Sakarya, Turkey; 2 Department of Bioistatistics, Faculty of Medicine, Sakarya University, Sakarya, Turkey

Dear Editor, After the coronavirus disease 2019 (COVID-19) pandemic affected the whole world, rheumatologists began to think about how COVID-19 will progress in patients with inflammatory conditions. High cytokine levels play a role in the pathophysiology of COVID-19 infection.

Tumor necrosis factor alpha (TNF-α) is a proinflammatory cytokine known to have a key role in the pathogenesis of chronic immune-mediated diseases. Anti-TNF therapy may cause an increase in active tuberculosis, other granulomatous diseases, and serious infections [1] . According to many studies, rheumatological diseases have not been identified as a risk factor for severe COVID-19 infection [2] . Should significantly increased cytokine levels during COVID-19 infection make us consider anti-cytokine therapies that may be used in the treatment of patients with COVID-19 a risk?

We aimed to explore whether the frequency of COVID-19 infection increased, the effect of comorbidities on the frequency of infection, and whether the severity of the disease and need for intensive care support increased in patients who used anti-TNF agents. We performed a retrospective case-control study between March and December 2020 in Sakarya University Training and Research Hospital. Retrospectively, we evaluated whether there was a difference in the frequency and severity of COVID-19 in our patients diagnosed with ankylosing spondylitis (AS), 77 of whom were using anti-TNF and 49 of whom didn’t use anti-TNF. Hospitalization and intensive care unit (ICU) requirements were evaluated as endpoints. In the anti-TNF group, patients used adalimumab, etanercept, certolizumab, infliximab, and golimumab. Patients were questioned at an outpatient clinic in person or by phone.

Seventy-seven patients with AS using anti-TNF agents (58 males, 19 females) and 49 patients with AS (38 males, 11 females) not using anti-TNF agents were included in the study (p = 0.943). Mean age of patients using anti-TNF agents was 41.53 ± 10.38, and mean age of patients not using anti-TNF agents was 42.94 ± 10.86 (p = 0.468). Thirty-three (42.9%) patients were smokers in the anti-TNF group, while 23 (46.9%) patients were smokers in the group not using TNFi (p = 0.791). There was 12 pack-year smoking in the anti-TNF group, and 14 pack-year smoking in not using TNFi (p = 0.623). The frequency of diabetes mellitus (DM), hypertension (HT), amiloidosis, familial mediterranean fever (FMF), coronary artery disease (CAD), chronic obstructive pulmonary disease (COPD) was similar in both groups (p = 0.403, p = 0.999, p = 0.521, p = 0.999, p = 0.999, respectively). Six patients using TNFi and 3 patients not using TNFi recovered from COVID-19 infection. However, this result was not statistically significant (p = 0.999). One patient using anti-TNF was hospitalized but with no need for admission to the ICU (p = 0.999). All 9 patients recovering from COVID-19 were male (p = 0.113). There were 2 (22.2%) smokers in the SARS-CoV-2 positive group and 54 (46.2%) smokers in SARS-CoV-2 negative group (p = 0.297). There was 37.5 pack-year smoking in SARS-CoV-2 positive group, and 12 pack-year smoking in SARS-CoV-2 negative group (p = 0.151). Nobody has comorbidities (DM, HT, amiloidosis, FMF, CAD, COPD) in SARS-CoV-2 positive group. There were patients with DM (5.1%), HT (15.4%), amiloidosis (1.7%), FMF (1.7%), CAD (0.9%) and COPD (0.9%) in SARS-CoV-2 negative group (p = 0.999, p = 0.356, p = 0.999, p = 0.999, p = 0.999, p = 0.999, respectively). Having comorbidities was not detected to be associated with frequency of COVID-19. 31 (40.3%) patients were using adalimumab, 25 (32.5%) patients were using etanercept, 13 patients were using (16.9%) certolizumab, 6 (7.8%) patients were using golimumab, and 2 patients (2.6%) were using infliximab in TNF group. Six patients using anti-TNF (2 adalimumab, 1 etanercept, 1 golimumab,2 infliximab) and 3 nonuser patients recovered from COVID-19 (p = 0.999). No statistically significant difference was found between SARS-CoV-2 positive and negative patients in terms of the types of anti TNF they used.

Patients were called in March 2020, and they were advised to terminate their anti-TNF therapy, when the COVID-19 pandemic began. Among those who used anti-TNF, 2 (33.3%) people who had COVID-19 and 38 (53.5%) people who did not have COVID-19 interrupted treatment (p = 0.419). Anti-TNF users who did not have COVID-19 stopped taking the treatment for an average of 3 months (min 2–max 4 months) starting from March 2020, and the patients who had COVID-19 (p = 0.102) stopped taking the treatment for 1.5 months (min 1–max 2 months). Duration of interrupting TNFi was not significant for the risk of COVID-19.

Comorbidities, older age, and the presence of active disease have been associated with worse outcomes in previous studies [3] . In our study, the anti-TNF using and the nonuser groups were similar according to age, sex, and comorbidities. Although comorbidities in COVID-19 are associated with severe disease in the literature, we did not find a significant difference in our study. This result is probably related to our insufficient number of patients. As a result, we found that the use of anti-TNF did not increase the frequency and severity of COVID-19. In a recently published multicenter study, it was stated that the use of biological DMARDs in patients with inflammatory rheumatic diseases was not significantly associated with a worse outcome of COVID-19. But unlike our study, having no comorbidities was associated with a decreased risk of a worse outcome [4].

There are currently studies investigating the therapeutic utility of infliximab and adalimumab in hospitalized COVID-19 patients [5] . The results of these studies are very important. The usability of TNFi in treatment and at which stage of the disease anti-TNF agents can be used are wondered. We will see the course of the disease all over the world after the administration of the COVID-19 vaccines, but we still need more information about effective and safe treatment.
